# Estimation of Axle Torque for an Agricultural Tractor Using an Artificial Neural Network

**DOI:** 10.3390/s21061989

**Published:** 2021-03-11

**Authors:** Wan-Soo Kim, Dae-Hyun Lee, Yong-Joo Kim, Yeon-Soo Kim, Seong-Un Park

**Affiliations:** 1 Department of Biosystems Machinery Engineering, Chungnam National University, Daejeon 34134, Korea; wskim0726@gmail.com (W.-S.K.); kimtech612@gmail.com (Y.-S.K.); 2 Department of Smart Agricultural Systems, Chungnam National University, Daejeon 34134, Korea; 3 Smart Agricultural Machinery R&D Group, Korea Institute of Industrial Technology (KITECH), Gimje 54325, Korea; 4 Reliability Test Team, TYM ICT Co. Ltd., Gongju 32530, Korea; psu0322@tymict.com

**Keywords:** agricultural tractor, axle torque, soil physical properties, multiple regression, artificial neural network

## Abstract

The objective of this study was to develop a model to estimate the axle torque (AT) of a tractor using an artificial neural network (ANN) based on a relatively low-cost sensor. ANN has proven to be useful in the case of nonlinear analysis, and it can be applied to consider nonlinear variables such as soil characteristics, unlike studies that only consider tractor major parameters, thus model performance and its implementation can be extended to a wider range. In this study, ANN-based models were compared with multiple linear regression (MLR)-based models for performance verification. The main input data were tractor engine parameters, major tractor parameters, and soil physical properties. Data of soil physical properties (i.e., soil moisture content and cone index) and major tractor parameters (i.e., engine torque, engine speed, specific fuel consumption, travel speed, tillage depth, and slip ratio) were collected during a tractor field experiment in four Korean paddy fields. The collected soil physical properties and major tractor parameter data were used to estimate the AT of the tractor by the MLR- and ANN-based models: 250 data points were used for developing and training the model were used, the 50 remaining data points were used to test the model estimation. The AT estimated with the developed MLR- and ANN-based models showed agreement with actual measured AT, with the R^2^ value ranging from 0.825 to 0.851 and from 0.857 to 0.904, respectively. These results suggest that the developed models are reliable in estimating tractor AT, while the ANN-based model showed better performance than the MLR-based model. This study can provide useful results as a simple method using ANNs based on relatively inexpensive sensors that can replace the existing complex tractor AT measurement method is emphasized.

## 1. Introduction

The transmission of a tractor is a complex system. It is one of the components that must have high reliability, as it is the most expensive of the various major components of the tractor [1,2]. The conventional tractor design method is reliant on the engine specifications or the weight of the tractor, rather than reflecting actual field load conditions [3]. Thus, load-sensing under actual agricultural working conditions is required to secure the reliability of the tractor transmission [4]. The axle torque (AT) of a tractor during agricultural operations is directly related to the transmission torque, which makes it possible to estimate the torque acting on all parts of the transmission by using the AT [5]. These torque data can be applied to achieve the optimal design of the transmission and can also be used as important data for carrying out various performance and durability tests, such as transmission endurance test [3]. Therefore, for optimal transmission design, AT data generated during agricultural operations under various conditions are required.

Many researchers have conducted studies on load measurement of the engine [6,7], transmission input axle [6], driving AT [3,8], and traction force [9]. Bai et al. [6] measured the engine and transmission output shaft torque of a multi-axle vehicle powertrain system, in order to analyze the influence of the main factors on the powertrain load dynamic characteristics and fatigue damage. Kumar et al. [10] developed a microcontroller based embedded system for measuring dynamic wheel axle torque and drawbar power of agricultural tractor for tillage research. They reported that, a maximum variation of ±320 Nm torque between the theoretically calculated and experimentally measured values under field load conditions. However, the above method has the disadvantage of having to install strain gauges on the transmission and drive axles, as well as having to build expensive telemetry systems, rather than wire-type sensors, as the axle rotates [5].

Tractor ATs can be estimated using relatively inexpensive sensors and multiple linear regression (MLR)-based models [11]. MLR-based models have been used to estimate dependent variables in various research fields, where the models are developed based on statistical analysis methods by adopting explanatory variables that are closely related to the prediction targets [12]. In general, it is important to select variables that have a great influence on the AT of a tractor, in order to develop MLR-based estimation models [13]. To date, many researchers have analyzed the major tractor parameters affecting the tractor AT. As a result, major parameters such as slip ratio (SR) [14,15], travel speed (TS) [11], and tillage depth (TD) [10] have been found to have a great influence on the tractor AT. As the driving axle of the tractor during agricultural operations interacts directly with the soil, the axle of the tractor is affected not only by the major tractor parameters, but also by the physical properties of the soil [16,17]. Therefore, some researchers have reported that the soil cone index (CI) [16,18,19] and soil moisture content (SMC) [17,20] influence the torque of the tractor axle. In conclusion, in order to apply a tractor AT estimation model, it is necessary to select appropriate variables, taking into consideration the ease of measurement and the associated correlation coefficients. However, in order to use such an MLR-based model, there is an associated inconvenience of considering the problem of multicollinearity between various variables and the linearity of variables.

Recently, various machine learning-based research considering techniques such as ANN, which have been shown to be strong in nonlinear analysis cases, has actively been conducted, having also been applied in agricultural research [9,21]. Research on engine torque (ET) estimation based on ANNs using data obtained through low-cost sensors as input variables has been reported by several researchers [22]. Bietresato et al. [22] proposed an ANN-based model using exhaust gas (EG) and motor oil temperature data as major variables to estimate the ET and brake-specific fuel consumption (BSFC) of a tractor. They reported that the ANN using EG temperature for torque estimation achieved a higher mean coefficient of determination (R^2^) than the ANN predicting BSFC in both the training and prediction stages. Rajabi-Vandechali et al. [23] proposed a tractor ET estimation model based on soft computing using a low-cost sensor. They estimated ET using two models, including a radial basis function (RBF) neural network and an adaptive neuro fuzzy inference system (ANFIS) and, as a result, ET could be estimated using engine speed (ES), fuel mass flow, and exhaust gas temperature. In addition, it was reported that the RBF outperformed ANFIS among the two models. The above studies suggest that the performance of various prediction models using ANNs based on low-cost sensors has been improving. Therefore, we expected such a model to be applicable to tractor AT estimation, as targeted in this study. In particular, the ANN-based model is expected to be expandable to a wider range, as it can also consider soil parameters, which are often non-linear variables, unlike studies that only consider existing tractor variables.

As mentioned above, an ANN can be used to develop estimation models with higher accuracy than conventional approaches. This suggests that this state-of-the-art technology can be applied to model development to estimate tractor AT using a relatively low-cost sensor. Therefore, in this study, we estimated the AT of the tractor as a function of soil physical properties and tractor major parameters using an ANN, comparing its ability to estimate the AT of the tractor with that of a model based on MLR. A simple method using ANN based on a relatively inexpensive sensor that can replace the traditional complex tractor AT measurement method is emphasized. Our approach contributes to the following key points: (1) It provides a simple algorithm for estimating tractor AT that can replace the need for expensive torque sensors, (2) We improve the performance of the model by developing an estimation model that considers not only linear variables but also nonlinear variables, (3) Various applications in agricultural machinery for realization of digital agricultural technologies such as real-time transmission failure diagnosis are possible.

## 2. Materials and Methods

### 2.1. Tractor

To measure field data, an agricultural tractor (S07, TYM Co. Ltd., Gongju, Korea) was used in this study. The engine rated power was 78 kW at the rated rotational speed of 2300 rpm. The transmission consisted of two power shifts, four driving shifts (of synchromesh type), and four range shifts (of constant mesh type). The tractor was equipped with a bias fly. The front and rear tires were 13.6–24 8PR and 18.4–34 10PR, respectively. Table 1 lists the detailed specifications of the used tractor.

### 2.2. Data Measurement

**Figure 1:**
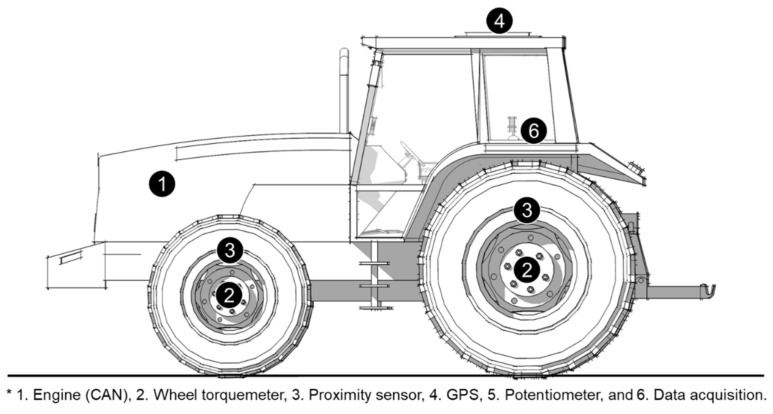
Load measurement system installed at each measurement position on the tractor.

Figure 1 shows the composition of the load measurement system installed at each measurement position on the tractor. The load measurement system consisted of: (a) Tier-4 electronic engine capable of CAN communication, (b) wheel torquemeter, (c) proximity sensor, (d) GPS, (e) potentiometer, and (f) data acquisition (DAQ). As the tractor was equipped with a Tier-4 electronic engine (D34P, Doosan Infracore Co., Ltd., Incheon, Korea), the major engine parameters could be obtained using the CAN communication framework. In this study, the major parameters of the engine were measured from the engine control unit (ECU) via the J1939 protocol. CAN message data, including ET, ES, and fuel consumption (FC), were measured during a field experiment with an accuracy of 1.0%. Rear wheel torquemeters (Manner Sensortelemetrie GmbH, MW 30 kNm Fu PCM16, Spaichingen, Germany) were mounted on each side, between the rear axles and wheels of the tractor, for measuring rear wheel torque. The torquemeters had a linearity deviation of 0.2%. The theoretical axle speed (AS)-sensing systems were located on each side, between the rear axles and wheels of the tractor. Each sensing unit was comprised of a proximity sensor (Autonics, PRDCMT30-25DO, Seoul, Korea) and a 100-gear tooth. The GPS (18 × 5 Hz, Garmin, Olathe, KS, USA), with an accuracy of 5.0%, was used to measure the actual TS of the tractor. The TD-sensing system was located on a 3 point-hitch lift arm. It was measured using a potentiometer built into the tractor (calibration of the potentiometer was performed in our previous work [17]). To measure the major parameters of the tractor, a DAQ (IMC, CRONOS compact CRC-400-11, Berlin, Germany) was used. The measured tractor performance parameters included:

- ET, in Nm;
- ES, in rpm;
- FC, in L·h^−1^;
- AT, in Nm;
- AS, in rpm;
- TS, in km·h^−1^; and
- TD, in cm.

Soil physical properties such as SMC and CI have a significant impact on tractor AT occurring during tillage [14,17]. Several previous studies have reported that the soil physical conditions may be different, even at the same site, as the soil conditions are generally non-uniform. Therefore, for reliable data collection, the field site was divided into a 3 m × 3 m grid to collect SMC and CI data [16,17]. SMC was measured using a soil moisture sensor (TDR350, Spectrum Technologies, Aurora, IL, USA) with two rods (each 20 cm in length), while the CI was measured using a 113 mm^2^ conical penetrometer (SC900, Spectrum Technologies) at a speed of about 30 mm·s^−1^ [24,25].

### 2.3. Data Collection

The tractor field experiment was conducted in March 2019 in a South Korean four-paddy field. The plow operation was performed at the M3-low gear stage (7.09 km·h^−1^); the most frequently used stage for plowing a 78 kW tractor, as determined from a user survey [26]. The engine rotational speed of the tractor was maintained at full throttle throughout the survey (2500 rpm). The TD was set to a range of approximately 15–20 cm and controlled by the occurrence of the workload through the depth control system mounted on the tractor. Plow tillage was performed using the C-type working pattern, which is commonly used by farmers in South Korea. 

### 2.4. Data Processing

The tractor overcame the workload, in the event of high loading, by lowering the ES to increase the torque based on the three-dimensional engine performance curve (ET-ES-FC). This is not only related to the engine rotational speed and torque, but also to the FC. Typically, the FC of the tractor is used as the SFC, which divides the engine power by the FC. The engine power was calculated using the torque and speed, based on Equation (1), which is a widely known method. The SFC was calculated according to Equation (2). The SR was calculated using the ratio of the theoretical speed to the actual TS using Equation (3). The specific gravity of diesel used in this study was 0.833 g·L^−1^. To develop an estimation model with significant variables, we used 250 data points, while the 50 remaining points were used to validate the model estimation:

(1) $$P=\frac{2\pi \mathrm{TN}}{60,000},$$

where P is the power (kW), T is the torque (Nm), and N is the rotational speed (rpm):

(2) $$\mathrm{SFC}=\frac{\mathrm{FC}}{P_{e}}\times \mathrm{SG},$$

where SFC is the specific fuel consumption (g·kWh^−1^), FC is the fuel consumption (L·h^−1^), $P_{e}$ is the engine power (kW), and SG is the specific gravity (g·L^−1^):

(3) $$s=\left(\frac{V_{0}-V_{a}}{V_{0}}\right)\times 100\left(\%\right),$$

where s is the slip ratio (%), $V_{0}$ is the theoretical speed (km·h^−1^), and $V_{a}$ is the travel speed (km·h^−1^).

The major field data used in this study are described using certain statistical parameters: The maximum, minimum, and average values, standard deviation (SD), and coefficient of variation (CV). The CV was calculated using Equation (4) [27]:

(4) $$\mathrm{CV}=\frac{\mathrm{SD}}{\mathrm{Average}}\times 100\left(\%\right),$$

where CV is the coefficient of variation (%) and SD is the standard deviation.

### 2.5. Multiple Linear Regression Analysis

Equation (5) represents a widely known MLR model [27]. In this study, the regression models were divided into four classes (cases 1, 2, 3, and 4), considering the convenience of data measurement. Case 1, which does not require the measurement of major soil physical properties, is based on the tractor engine parameters (ET, ES, and SFC). Case 2 considers three major tractor parameters (TD, SR, and TS) that affect the AT, in addition to the tractor engine parameters. Case 3 considers two soil physical properties (SMC and CI), in addition to the tractor engine parameters. Case 4 used all of the input variables, including the three engine parameters, three tractor major parameters, and two soil physical properties:

(5) $$Y_{i}=a_{0}+a_{1}x_{1}+a_{2}x_{2}+\cdots +a_{n}x_{n}+\varepsilon ,$$

where $Y_{i}$ is the value of the response variable for the ith case; $a_{0},a_{1},a_{2}\cdots a_{n}$ are the coefficients of the multiple regression; $x_{1},x_{2}\cdots x_{n}$ are the regression model variables; and ε is the error of the model.

### 2.6. Artificial Neural Networks (ANNs)

ANNs were designed using biological inspiration from the interconnection of neurons in the brain. A perceptron, which is mathematical model of simplified neuron, is the basic component in an ANN, is the basic component, which works as linear classifier through weighted summation and the activation of input signals. An ANN can solve regression and classification tasks efficiently, by connecting perceptron hierarchically and densely. These stacked structures have the potential to solve the complex regression problems, even those with non-linear characteristics [28]. Neural networks (NN) consist of input, hidden, and output layers, where deeper hidden layers may allow the network to better fit to the training data, at the cost of decreasing generalization ability for field or test data. For this reason, NN-based regression models mostly use two hidden-layers, such as those previously designed for draft force prediction [9], constituent properties estimation [21], and Carcass weight prediction [13]. Thus, a two-hidden layer NN architecture was adopted to estimate the AT for the agricultural tractor in this study. The number of neurons in each hidden layer was set experimentally to 32 and 16, respectively that have minimum size to train the training set in which the loss is saturated to zero. Batch normalization (BN) was added following each hidden layer, in order to improve training efficiency and to prevent the vanishing gradient problem. The sigmoid function was added after BN, as the activation function. A total of eight input variables—including three engine parameters, three major tractor parameters and two soil physical properties—were used, along with the measured AT as training data, where 200, 50, and 50 data points were used for network training, validation, and testing, respectively. Each variable had a different scale, making the training process slow and potentially leading the model to become stuck in local minima. Thus, the variables were normalized (by scaling between 0 and 1) using min-max normalization as in Equation (6) during the model training stage. The loss function consisted of the mean squared error (MSE) with a regularization penalty term (L2 regularization with 0.001 lambda). The weights in the NN were updated iteratively using a gradient descent optimizer with 0.9 momentum. Model training was stopped when the validation loss had the lowest value, in order to avoid overfitting to the training data. The validation loss was calculated for every training iteration by using the loss function with 50 validation data points:

(6) $$X_{\mathrm{norm}}=\frac{x-\min\left(x\right)}{\max\left(x\right)-\min\left(x\right)},$$

where $X_{\mathrm{norm}}$ is the normalized value, x is the original value, $\min\left(x\right)$ is the minimum value, $\max\left(x\right)$ is the maximum value. 

### 2.7. Software

The MLR analysis was developed using the SPSS 21 software (SPSS Inc., Chicago, IL, USA), while Python (ver. 3.7, Python Software Foundation, Wilmington, DE, USA) and Pytorch (ver. 4.0, Berkeley Software Distribution, San Mateo, CA, USA) were used to implement the ANN.

### 2.8. Performance Evaluation Parameters

All proposed estimation models, including the MLR and ANN models, were verified by comparing the AT measured in the actual field, based on a 1:1 line. In this study, the performance evaluation of the developed estimation model was performed using statistical parameters such as coefficient of determination (R^2^), mean absolute percentage error (MAPE), root mean square error (RMSE), and relative deviation (RD), as shown in Equations (7)–(10):

(7) $$R^{2}=\frac{\sum_{i=1}^{i=N}\left(y_{i}-y_{a}\right)-\sum_{i=1}^{i=N}\left(y_{i}-\hat{y_{i}}\right)}{\sum_{i=1}^{i=N}\left(y_{i}-y_{a}\right)},$$

(8) $$\mathrm{MAPE}=\frac{1}{N}\overset{i=N}{\underset{i=1}{\sum }}\left|\frac{1}{y_{i}}\left(y_{i}-\hat{y_{i}}\right)\right|\times 100\left(\%\right),$$

(9) $$\mathrm{RMSE}=\sqrt{\frac{1}{N}\overset{i=N}{\underset{i=1}{\sum }}{\left(\hat{y_{i}}-y_{i}\right)}^{2}},$$

(10) $$\mathrm{RD}=\frac{\mathrm{RMSE}}{\mathrm{Mean}}\times 100\left(\%\right),$$

where $y_{a}$ is the mean measured AT, $y_{i}$ is the ith measured AT, and $\hat{y_{i}}$ is the ith estimated AT.

## 3. Results

### 3.1. Data Analysis 

**Figure 2:**
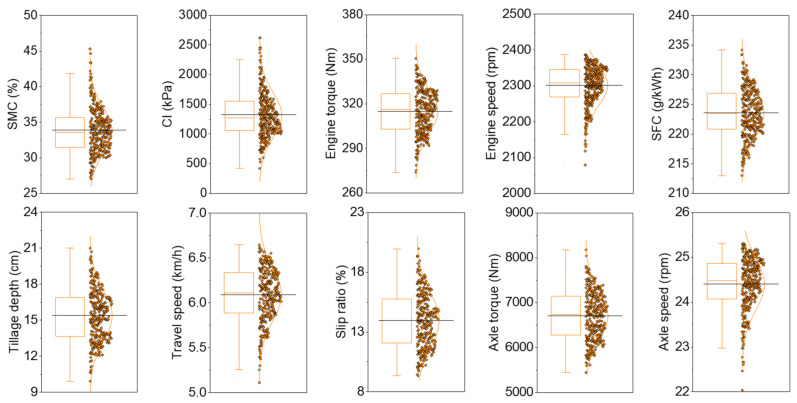
Boxplot analysis of the soil physical properties and major tractor parameters.

Figure 2 shows a boxplot of the analysis results of the soil physical properties and major tractor parameters. The line across the box represents the median. The boxes represent the 25th and 75th percentiles. The whiskers represent the maximum and minimum values of each data, excluding outliers. In the total data, most of the variables appeared to be normally distributed, and the median and mean values were found to be similar. On the other hand, the ES and AS were skewed toward higher values. This was because, in most cases, the engine operates at the set ES condition; however, in some cases, the ES decreases as the engine outputs a high load by throttling down at some points that require a high load for the tillage operation. As the AS is related to the ES, the data distribution of these two variables was considered to be due to the same cause.

Table 2 provides the statistical description of the data, with outliers removed based on the data measured in the field. The SMC and CI showed distributions of 27–45% and 422–2620 kPa, respectively. The CVs of SMC and CI were 9.4 and 29.0%, respectively, showing high volatility. The ranges of ET, ES, and SFC were 274–351 Nm, 2079–2387 rpm, and 213–234 g·kWh^−1^, respectively, with CV lower than 5%. The TD was in the range of 9.9–21.0 cm, with high CV at 13.8%. This can differ, in part, due to soil elevation, and is considered to be related to the work load. The TS and SR ranged between 5.11–6.65 km·h^−1^ and 9.3–20.0%, respectively, with CVs of 4.9% and 16.4%, respectively. As the tractor operated in irregular soil conditions, the SR was considered to be high, as it was affected by the physical properties of the soil. The AT and AS ranged between 5449–8180 Nm and 22.0–25.3 rpm, respectively, with CVs of 7.9% and 2.4%, respectively. Overall, the highest variability in soil physical properties was observed and, among the major tractor parameters, high variability was observed for TD and SR.

### 3.2. Correlation Analysis

**Figure 3:**
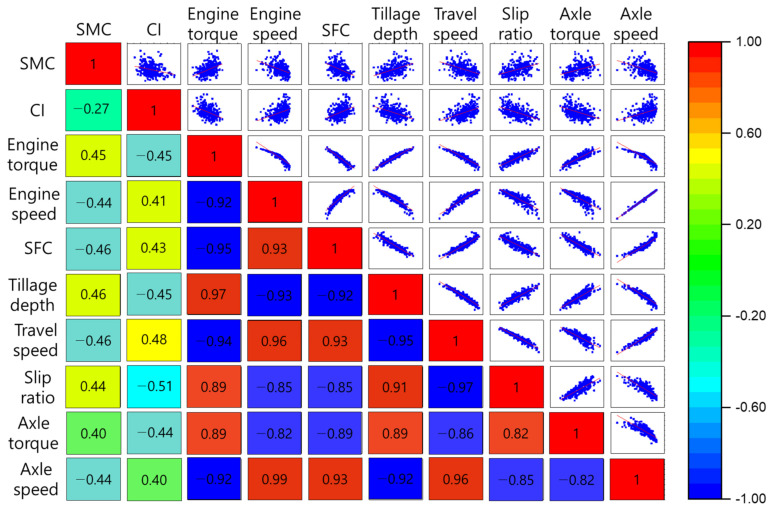
Correlation matrix plot between soil physical properties and major tractor parameters.

Figure 3 shows the correlation matrix between major tractor parameters and soil physical properties. The upper triangular section of the matrix shows bivariate scatter plots with fitted lines. The lower triangular section of the matrix shows the Pearson correlation coefficients. The correlation coefficients between all variables had a significance level of *p* < 0.01. The correlation coefficients between soil physical properties and major tractor parameters showed a minimum of 0.4 (SMC and AT; CI and AS) and maximum of −0.51 (CI and SR). The correlation coefficient between SMC and CI, which are the soil physical properties, was −0.27, which means that the effects between the variables were low. In contrast, the correlation coefficients between the major sensor data of the tractor were higher than 0.82 (or less than −0.82). This means that, when the tractor was driven to overcome the high load required for soil destruction during plowing, all data were highly correlated. The lowest correlation coefficients among the tractor variables were between ES and AT (r = −0.82), AT and AS (r = −0.82), and SR and AT (r = 0.82). The highest correlation coefficient was between ES and AS (r = 0.99). ES and AS are directly connected by driving shafts and, unlike torque, power transmission efficiency is not applied and is calculated through the simple gear ratio, thus showing a highly positive correlation coefficient. The variables that had the greatest influence on AT were ET (r = 0.89), SFC (r = −0.89), and TD (r = 0.89). 

### 3.3. Estimation of Tractor Axle Torque Using Multiple Linear Regression

To develop the MLR-based model, the variables used for the AT estimation model development were the two soil physical properties and seven major tractor parameters. The significant variables in the model were similar to that of a previous analysis of tractor traction load [9] and AT [11], where ES, TD, SMC, and CI were the common factors in estimating the AT. Equations (11)–(14) are the MLR-based models using variables corresponding to the cases 1, 2, 3, and 4 mentioned above, respectively:

(11) $$T_{a}=17.22\mathrm{ET}+2.748\mathrm{ES}-93.19\mathrm{SFC}+15,805,$$

where $T_{a}$ is the axle torque (Nm), ET is the engine torque (Nm), ES is the engine speed (rpm), and SFC is the specific fuel consumption (g·kWh ^−1^):

(12) $$T_{a}=-5.398\mathrm{ET}+3.036\mathrm{ES}-111\mathrm{SFC}+555\mathrm{TS}+180\mathrm{TD}+2953\mathrm{SR}+19,740,$$

where TS is the travel speed (km·h^−1^), TD is the tillage depth (cm), and SR is the slip ratio (%):

(13) $$T_{a}=16.34\mathrm{ET}+2.633\mathrm{ES}-93.728\mathrm{SFC}-3.739\mathrm{SMC}-0.063\mathrm{CI}+16,676,$$

where SMC is the soil moisture content (%) and CI is the cone index (kPa):

(14) $$T_{a}=-6.316\mathrm{ET}+2.904\mathrm{ES}-113\mathrm{SFC}+563.1\mathrm{TS}+185.1\mathrm{TD}+2542\mathrm{SR}-6.588\mathrm{SMC}-0.059\mathrm{CI}+20,955.$$

**Figure 4:**
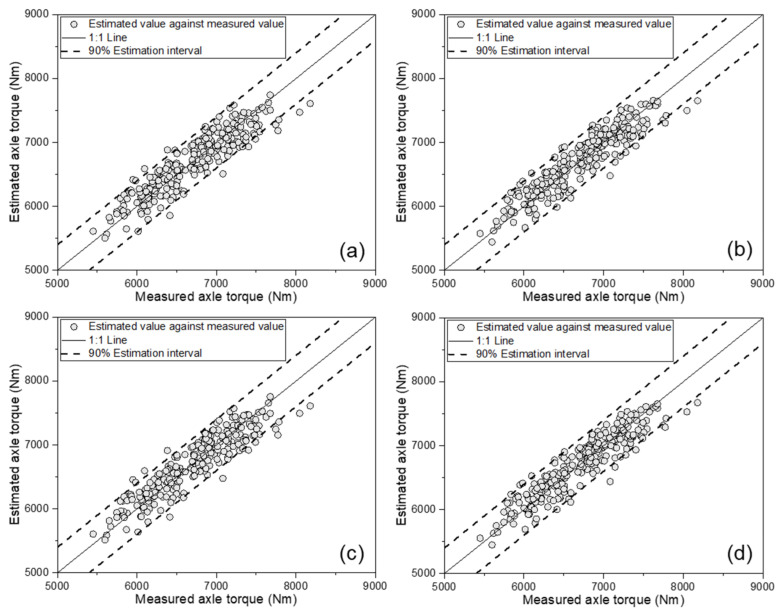
Results of MLR-based axle torque estimation models, comparing estimated values against measured axle torque: (**a**) As a function of the engine parameters; (**b**) as a function of the engine parameters and major tractor parameters; (**c**) as a function of the engine parameters and soil physical properties; and (**d**) as a function of the engine parameters, major tractor parameters, and soil physical properties.

Figure 4 shows the results of the MLR-based AT estimation model, comparing the measured and estimated ATs, according to the four different cases. As a result of model development, in all four cases, the estimated AT based on a 1:1 line well-described the measured AT. Table 3 listed major results of the calibration set of the AT estimation model based on MLR. The model using the tractor engine parameters (ET, ES, and SFC) as input variables showed an R^2^ of 0.825. The model considering not only three tractor engine parameters, but also the major tractor parameters TS, TD, and SR showed an R^2^ of 0.849. In addition, the model with the two soil physical parameters SMC and CI, in addition to the three tractor engine parameters, showed an R^2^ of 0.827. Therefore, the major tractor parameters had a greater effect on improving the performance of the model than the soil physical properties. Finally, the model considering all eight variables showed the highest R^2^ of 0.851.

**Figure 5:**
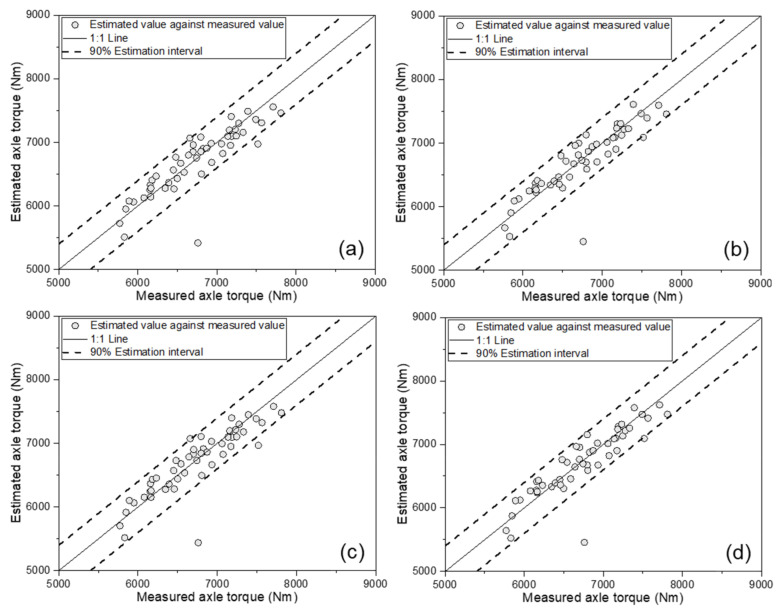
Results for MLR-based axle torque estimation model validation, comparing estimated values against measured axle torque: (**a**) as a function of the engine parameters; (**b**) as a function of the engine parameters and major tractor parameters; (**c**) as a function of the engine parameters and soil physical properties; and (**d**) as a function of the engine parameters, major tractor parameters, and soil physical properties.

Figure 5 shows the MLR-based AT estimation model verification results, comparing the measured AT and the estimated value. In all four estimation models, most of the estimated data points were located within the 90% estimation interval line, while only a few points were outside the 90% estimation interval line. Table 4 lists the results of the performance evaluation on the validation set of the MLR-based AT estimation model. All four models were found to have an R^2^ of 0.75 or more, a MAPE within 2.8%, a RMSE of less than 270 Nm, and a RD of less than 4.0%. Among them, the last model—the one using all eight variables (ET, ES, SFC, TS, TD, SR, SMC, and CI)—showed the highest performance. This model achieved an R^2^ of 0.775, an MAPE of 2.58%, an RMSE of 255 Nm, and an RD of 3.8%, showing the best performance. Thus, these models can be considered applicable for estimating the AT of the tractor.

### 3.4. Estimation of Tractor Axle Torque Using Artificial Neural Network

**Figure 6:**
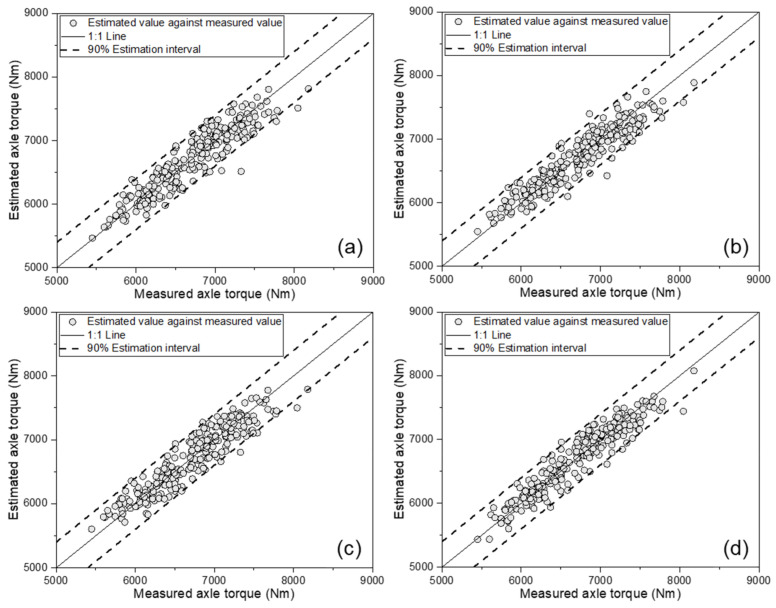
Results for ANN-based axle torque estimation model development, comparing estimated values against measured axle torque: (**a**) as a function of the engine parameters; (**b**) as a function of the engine parameters and major tractor parameters; (**c**) as a function of the engine parameters and soil physical properties; and (**d**) as a function of the engine parameters, major tractor parameters, and soil physical properties.

Figure 6 shows the results of the ANN-based AT estimation model, comparing the measured AT and estimated values, according to the various input variables. In all four cases, the estimated AT based on a 1:1 line well-described the measured AT. Table 5 lists the major results on the calibration set of the ANN-based AT estimation model. Basically, the model with tractor engine parameters (ET, ES, and SFC) as input variables had an R^2^ of 0.857. In addition to the three engine parameters, the model considering the three major tractor parameters (TS, TD, and SR) showed an R^2^ of 0.885, while the R^2^ of the model considering soil physical properties (SMC and CI) in addition to the engine parameters was 0.875. In the model of the most complex system—that considered all of the engine parameters, major tractor parameters, and soil physical properties—the R^2^ was 0.904, thus showing the best performance.

**Figure 7:**
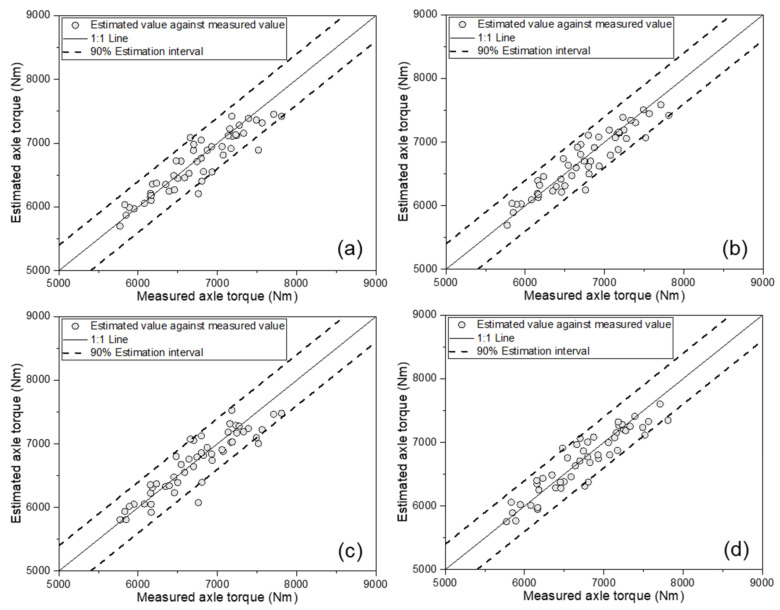
Results of ANN-based axle torque estimation model validation, comparing estimated values against measured axle torque: (**a**) as a function of the engine parameters; (**b**) as a function of the engine parameters and major tractor parameters; (**c**) as a function of the engine parameters and soil physical properties; and (**d**) as a function of the engine parameters, major tractor parameters, and soil physical properties.

The developed models were verified using the data of the remaining 50 data points which were not used for model training. This verification step made it possible to verify the generalization performance of the developed ANN-based models. Figure 7 shows the verification results of the developed ANN-based models, comparing the measured and estimated AT. For all four estimation models, most data points were located within the 90% estimation interval. In particular, when all variables were used, as shown in Figure 7d, all data points were located within the 90% line. Table 6 lists the performance on the validation set for the ANN-based AT estimation models. The model using only the tractor engine parameters showed good performance, with an R^2^ of 0.841, MAPE of 2.36%, RMSE of 215 Nm, and RD of 3.21%. The model using the tractor engine parameters and major tractor parameters showed the highest performance, with an R^2^ of 0.870, MAPE of 2.25%, RMSE of 192 Nm, and RD of 2.87%. Among the four cases, the third model, which used the tractor engine parameters and soil physical properties, showed the lowest performance, with an R^2^ of 0.821, MAPE of 2.53%, RMSE of 224 Nm, and RD of 3.35%. The final model, which used all eight variables, showed the second-best performance among the four models, with an R^2^ of 0.847, MAPE of 2.48%, RMSE of 207 Nm, and RD of 3.10%. All four models were found to have an R^2^ of 0.82 or more, a MAPE within 2.6%, a RMSE of less than 230 Nm, and a RD of less than 3.4%. Unlike the calibration set, in the validation set, the model that considered major tractor parameters in addition to the tractor engine parameters showed the highest performance. This was a result that may have appeared due to verification using data that were not used for model development and, in case 4—in which all variables were used for the model—it was judged that the performance in the validation set was slightly lower due to issues such as overfitting of the model in the calibration set. Nevertheless, this was considered to be acceptable, as the overall performance of all models was found to be excellent. Therefore, these four models can be considered applicable for estimating the tractor AT.

### 3.5. Comparison of Axle Torque Estimation Model by MLR and ANN 

**Figure 8:**
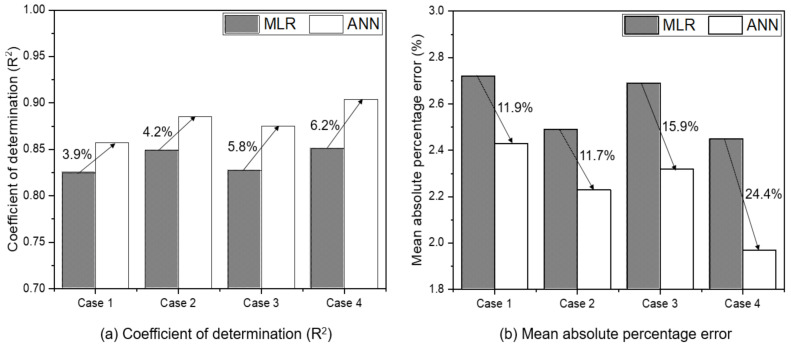
Results of comparison of tractor axle torque estimation performance by MLR- and ANN-based models: Case 1 used engine parameters (ET, ES, and SFC) as input variables. Case 2 used engine parameters and major tractor parameters (TS, TD, and SR) as input variables. Case 3 used engine parameter and soil physical properties (SMC and CI). Case 4 used engine parameters, major tractor parameters, and soil physical properties.

Figure 8 shows the results of comparison for the tractor AT estimation performance, according to the MLR- and ANN-based models, in the calibration set. Overall, the R^2^ improved by 3.9–6.2% in the ANN-based models, compared to the MLR-based ones, as shown in Figure 8a. Case 1, which used only the tractor engine variables—the most basic variable—showed the lowest improvement rate of 3.9%. Case 2, where the three major tractor parameters were added to those used in Case 1, showed the second lowest improvement rate of 4.2%. On the other hand, Case 3, which added the two soil physical variables as main variables to Case 1, showed an improvement rate of 5.8%, which was superior to that of Case 2. Case 4, which considered all variables, showed the highest improvement rate of 6.2%. Figure 8b shows the comparison results of MAPE for the MLR- and ANN-based models. Overall, the MAPE was about 11.7–24.4% lower in ANN, compared to MLR. Similar to the R^2^ results, Case 3, which adopted soil physical properties as variables, showed a higher MAPE reduction rate than Case 2, which adopted the main tractor parameters as variables. Case 4, using all variables, showed a decrease of 24.4% in ANN, compared to MLR, showing the greatest difference according to the two different methods. In all cases, ANN showed better performance than MLR. In particular, in case 3 and case 4, ANN showed an increase in R2 of 5% or more and a decrease in MAPE of 15% or more. In case 3 and case 4, a soil physical properties were adopted as an input variable, and this results showed that ANN would be better compared to MLR when using soil physical properties which is somewhat nonlinear characteristics as input variables as in the previous studies [29].

## 4. Discussion

In this study, tractor AT estimation models based on MLR and ANN were proposed, and the major results (by each of the four input cases) were compared. Overall, the MLR-based models had R^2^ values of 0.825–0.851, while the ANN-based models had R^2^ of 0.857–0.904. These results were found to be similar to the main results of previous studies using ANNs in agricultural research: Yield prediction of winter rapeseed (R^2^ = 0.69) [30], draft force of a chisel cultivator (R^2^ = 0.94) [9], and constituent properties of Red apples (R^2^ = 0.738–0.923) [21].

As a result of the analysis according to the two modeling methods (i.e., MLR and ANN), the ANN-based model showed better performance. This was considered to be because the ANN is based on multi-layer perceptron and, as it can learn the relationships between measured data using a calibration set, the higher the number of input variables and the higher the dimension, the better the performance. In particular, in Cases 1 and 2, using only the tractor engine and main tractor variables (which are linear variables), the difference in R^2^ between MLR- and ANN-based methods was 3.9–4.2%. On the other hand, in Cases 3 and 4, using the soil physical properties (i.e., non-linear variables), the difference in R^2^ was 5.8–6.2%. Thus, it was found that, when a non-linear variable (e.g., SMC and CI, in this study) was used as an input variable, the performance of the ANN-based model was superior to that of the MLR-based model.

In our ANN-based model, the performance of the model on the calibration set was highest in Case 4, which used all variable conditions as input variables. On the other hand, in the validation set, the performance of model in Case 4 was the second highest, while the model in Case 2—which used engine parameters and major tractor parameters as input variables—showed the highest performance. These results are believed to be due to the high input dimensions and high model complexity of the estimation model. This means that the higher the input dimensions can make the model more fit to training data, while the generalization performance is degraded in the same model capacity although the model was trained to avoid overfitting by validation loss check for each case. As a result, the generalization performance of the model was considered to be poor. Nevertheless, it showed high performance in the calibration set, which means that the model capacity was sufficiently fit for the data; thus, it can be seen that the model can be considered to be applicable for tractor AT estimation.

## 5. Conclusions

In this study, we estimated tractor AT as a function of soil physical properties and major tractor parameters using two different types of modeling approaches. The MLR-based models had R^2^ values of 0.825–0.851 and the results of the model verification showed a MAPE of 2.58–2.73%. Meanwhile, the ANN-based models had R^2^ values of 0.857–0.904 and the results of the model verification showed a MAPE of 2.25–2.53%; however, depending on the input variables used, the performance of the model varied greatly. Comparison of the performance of MLR- and ANN-based methods revealed that the most important factor in increasing the R^2^ in ANN (compared to MLR) was the use of soil physical condition variables. These soil physical variables are non-linear and, thus, the influence of the soil physical properties was greater in ANN-based modeling than in basic MLR-based modeling. The main result of this study was that the ANN-based methods showed a better estimation performance than the MLR-based methods. Therefore, it is possible to estimate tractor AT using ANN.

This study is expected to provide a simple algorithm for estimating tractor AT, which can replace the need for expensive torque sensors and can be applied to the development of an automated system for predicting the fatigue life of a tractor transmission. Although these contribution, this study has several limitations as follows: (1) only general ANN architecture was used without consideration of various topologies like a non-iterative neural-like structure that can train faster [31], (2) since the variable conditions are very diverse, not all variable conditions affecting tractor AT in this study were considered, and (3) in this study, only 300 soil physical condition and major tractor parameter data measured in specific conditions were used. These issues will be addressed by applying various analysis techniques and collecting data through field experiments under various working conditions in future study.

In addition, the important issues learned through this study are as follows: In order to improve the performance of the tractor AT estimation model, it is necessary to consider even the soil physical properties, which are a nonlinear variable. While this can improve model performance, there is a risk of overfitting, which can lower the generalization performance. Therefore, both the addition of nonlinear variables and the risk of overfitting must be considered.

Finally, in this study, we proposed a method for applying the latest modeling technology to tractor AT estimation; we believe that various applications in this field will be possible in the future, for the realization of potentially various digital agriculture technologies.

## Figures and Tables

**Table 1:** Specifications of 78 kW agricultural tractor used in this study.

Item		Specifications
Dimension		4225 × 2140 × 2830 mm
Weight	Empty	39,093 N
	Total	51,071 N
Engine	Rated torque	324 Nm @ 2300 rpm
	Maximum torque	430 Nm @ 1400 rpm
Transmission	Type	Power Shuttle
	Number of gear stages	32 Forward/32 Reverse

**Table 2:** Statistical analysis results of soil physical properties and major tractor parameters.

Parameters	Max.	Min.	Median	Avg. ± SD	CV (%)
Soil moisture content (%)	45	27	34	43 ± 3	9.4
Cone index (kPa)	2620	422	1270	1322 ± 384	29.0
Engine torque (Nm)	351	274	316	315 ± 15	4.7
Engine speed (rpm)	2387	2079	2308	2301 ± 55	2.4
Specific fuel consumption (g·kWh ^−1^)	234	213	223	224 ± 4	1.8
Tillage depth (cm)	21.0	9.9	15.4	15.4 ± 2.1	13.8
Travel speed (km·h ^−1^)	6.65	5.11	6.11	6.09 ± 0.3	4.9
Slip ratio (%)	20.0	9.3	14.0	14.0 ± 2.3	16.4
Axle torque (Nm)	8180	5449	6741	6712 ± 532	7.9
Axle speed (rpm)	25.3	22.0	24.5	24.4 ± 0.6	2.4

**Table 3:** Performance on the calibration set for MLR-based axle torque estimation models.

Parameters	R^2^	MAPE (%)	RMSE (Nm)	RD (%)
ET + ES + SFC	0.825	2.72	223	3.33
ET + ES + SFC + TS + TD + SR	0.849	2.49	207	3.09
ET + ES + SFC + SMC + CI	0.827	2.69	222	3.31
ET + ES + SFC + TS + TD + SR + SMC + CI	0.851	2.45	206	3.07

**Table 4:** Performance on the validation set for MLR-based axle torque estimation models.

Parameters	R^2^	MAPE (%)	RMSE (Nm)	RD (%)
ET + ES + SFC	0.751	2.73	268	3.99
ET + ES + SFC + TS + TD + SR	0.772	2.61	256	3.82
ET + ES + SFC + SMC + CI	0.758	2.67	264	3.93
ET + ES + SFC + TS + TD + SR + SMC + CI	0.775	2.58	255	3.80

**Table 5:** Performance on the calibration set for the ANN-based axle torque estimation models.

Parameters	R^2^	MAPE (%)	RMSE (Nm)	RD (%)
ET + ES + SFC	0.857	2.43	205	3.05
ET + ES + SFC + TS + TD + SR	0.885	2.23	189	2.82
ET + ES + SFC + SMC + CI	0.875	2.32	194	2.89
ET + ES + SFC + TS + TD + SR + SMC + CI	0.904	1.97	170	2.54

**Table 6:** Performance on the validation set for ANN-based axle torque estimation models.

Parameters	R^2^	MAPE (%)	RMSE (Nm)	RD (%)
ET + ES + SFC	0.841	2.36	215	3.21
ET + ES + SFC + TS + TD + SR	0.870	2.25	192	2.87
ET + ES + SFC + SMC + CI	0.821	2.53	224	3.35
ET + ES + SFC + TS + TD + SR + SMC + CI	0.847	2.48	207	3.10

## Data Availability

Not applicable.
